# Chloroform Extract from Fermented *Viola mandshurica* Regulates LPS-Induced Inflammation Response in RAW 264.7 Cells by Inhibiting iNOS and COX-2

**DOI:** 10.4014/jmb.2408.08047

**Published:** 2024-12-12

**Authors:** Hyunju Kim, Kyoung-Sook Kim, Young-Choon Lee, Jong Hyun Cho

**Affiliations:** Department of Medicinal Biotechnology, College of Health Sciences, Dong-A University, Busan 49315, Republic of Korea

**Keywords:** COX-2, fermented *Viola mandshurica* (Violaceae), MAPK, NF-κB, iNOS

## Abstract

Inflammatory is a crucial part of the immune system of body protect it from harmful invaders, such as bacteria, viruses, and other foreign substances. In this study, the effects of chloroform extract of fermented *Viola mandshurica* (CEFV) on lipopolysaccharide (LPS)-induced inflammatory response in RAW264.7 macrophages were investigated. The CEFV significantly inhibited NO production and reduced the expression of inducible nitric oxide synthase (iNOS) at both protein and mRNA levels in a dose-dependent manner. Also, CEFV decreased PGE_2_ production, suppressed COX-2 expression, and inhibited the activation of the ERK and JNK pathways but not the p38 pathway. Taken together, CEFV suppressed NF-κB activation, which is a key regulator in the inflammatory response. The main phenolic compounds identified in CEFV were tectoridin, luteolin, resveratrol, and hesperetin. Therefore, in this study, CEFC exhibits potent anti-inflammatory effects by downregulating the production of pro-inflammatory mediators and inhibiting key inflammatory pathway in RAW264.7 cells.

## Introduction

Inflammation is a normal physiological phenomenon that occurs in response to microbial infection, trauma, or injury such as heat and radiation [1]. Macrophages are well known as inflammatory cells that are important in the initiation of inflammatory responses. They secrete many inflammatory mediators and inflammatory cytokines, playing an important role in the pathogenesis of inflammatory disease processes [2]. Lipopolysaccharide (LPS), a gram-negative bacterial outer membrane chemical, plays an important role in inducing inflammatory responses and causing various inflammatory diseases [3]. The interaction between LPS and toll-like receptor (TLR) 4 leads to the activation of intracellular signaling via the myeloid differentiation factor (MyD) 88 pathway, which is known to activate the major translocation of nuclear transcription factor κB (NF-κB) and mitogen-activated protein kinase (MAPK) (including p38, ERK1/2, and JNK), thereby inducing the transcription of specific genes, thereby regulating inflammatory responses [4].

Various plant natural products have increasing application as alternative medicines in various health and disease conditions [5]. According to WHO, more than 20,000 medicinal plants have been collected, and some plant-based natural products are also used as nutraceuticals [6, 7] In addition, potential secondary metabolites isolated from medicinal plants are used for pharmaceutical purposes, and approximately 50% of modern medicines are derived from these products [8].

Fermentation can break down complex compounds in medicinal plants and herbs into simpler forms, making the nutrients and bioactive compounds more bioavailable. During fermentation, microorganisms can metabolize the original compounds in the extract, leading to the production of new bioactive metabolites. These metabolites may have unique physiological functions that differ from those of the unfermented extract [9-11]. Utilizing fermented plant extracts as a source of anti-inflammatory compounds is of interest due to their potential natural and holistic benefits. Previous studies have shown that fermented herbal extracts have anti-inflammatory properties due to the transformation of certain compounds during fermentation [12-14]. It is well documented that the numbers of plant extracts have wildly anti-inflammatory properties. For example, genistein, flavonoid, reduced inflammation and destruction of joints induced by collagen in arthritic mice. And rutin, hesperidin, and quercetin reduced acute and chronic inflammation in an experimental model. Especially, tartary buckwheat sprout (TBS) containing a high concentration of rutin reduced NO production by inhibiting iNOS and COX2 expression in Lipopolysaccharide (LPS)-induced RAW 264.7 cells [15].

*Viola mandshurica*, a perennial herb in the family *Violaceae*, is a traditional herbal medicine with various pharmaceutical functions, including that of an expectorant, a diuretic, and anti-inflammatory against bronchitis, rheumatism, skin eruptions, and eczema [16]. Additionally, *V. mandshurica* has demonstrated significant antioxidant, anti-diabetic, and anti-asthmatic properties [17]. Also, in a previous study, we have demonstrated that chloroform extract from fermented *V. mandshurica* (CEFV) possesses strong inhibitory effects on melanogenesis induced by α- melanocyte-stimulating hormone (MSH) through inhibition of the phosphorylation of cAMP responsive element binding protein (CREB) and activation of extracellular signal-regulated kinase (ERK) in B16 melanoma cells [18]. However, *V. mandshurica* has not been investigated for anti-inflammatory activity. Therefore, we investigated the inflammatory response by CEFV treatment in a macrophage cell line stimulated by LPS.

## Materials and Methods

### Chemicals and Antibodies

Dulbecco's Modified Eagle's Minimum essential medium (DMEM), fetal bovine serum (FBS), penicillin, and streptomycin were obtained from Thermo Fisher Scientific Hyclone (USA). 3-(4,5-dimethylthiazol-2-yl)-2,5-diphenyltetrazolium bromide (MTT), L-N6-(1-iminoethyl) lysine (L-NIL), NS-398, lipopolysaccharide (LPS)(*Escherichia coli*, serotype 0111: B4), Triton X-100, and all other chemicals were purchased from the Sigma Chemical Co. (USA). RIPA buffer, Protease and Phosphatase Inhibitor Cocktail were obtained from Pierce (USA). NE-PER reagents were used to isolate nuclear and cytoplasmic extract from Thermo Fisher Scientific. Antibodies for COX-2, iNOS, p-ERK, ERK, p-p38, p38, p-JNK, JNK and horseradish peroxidase-conjugated secondary antibody were purchased from Santa Cruz Biotechnology Inc. (USA). Glyceraldehyde-3-phosphate dehydrogenase (GAPDH) antibody was purchased from Millipore Corporation (USA). The enzyme immunoassay (EIA) kit for PGE_2_ was obtained from Cayman (USA) and BioLegend (USA), respectively. The oligonucleotide primers of iNOS, COX-2, and β-actin were purchased from Bioneer (Republic of Korea).

### Preparation of CEFV

CEFV was prepared as described previously [18]. Briefly, the whole plant of *V. mandshurica* was mixed gently with crude sugar (half weight of the plant), packed them fully in a ceramic pot, and then fermented for 6 months in a cool dark place. To identify the major compound possessing the anti-inflammatory activity, fermented *V. mandshurica* extract (10 L) was diluted with three volumes of distilled water and partitioned with *n*-hexane (12 g), CHCl_3_ (1.5 g), EtOAc (8.7 g), BuOH (34 g).

### High Performance Liquid Chromatography (HPLC) Analysis of CEFV

Twenty-nine phenolic compound standards, flavonoids as catechin, naringin, naringenin, myricetin, quercetin, biochanin A, formononetin, hesperetin, kaempferol, rutin, gallic acid, pyrogallol, protocatechuic acid, gentisic acid, p-hydroxybenzoic acid, chlorogenic acid, vanillic acid, caffeic acid, syringic acid, luteolin, cinnamic acid, p-coumaric acid, ferulic acid, veratric acid, salicylic acid, benzoic acid, tectoridin, O-coumaric acid, and resveratrol were purchased from Sigma Aldrich (USA). Extrasynthese (rance) used for calibration curves. The standard stock solutions (50, 100, 250, and 500 ppm) were made with dimethylsulfoxide (DMSO). All standard calibration curves showed high degrees of linearity (r^2^ > 0.99) (data not shown). Sample compounds were identified on the basis of the retention times of standard materials and were quantified by comparing their peak areas with those of standard curves. Samples preparation for analysis of phenolic compounds was performed as described previously [17]. One gram of freeze-dried CEFV powder was mixed with 10 ml of acetonitrile and 2 ml of 0.1 N hydrochloric acid and stirred for 2 h at room temperature. The suspension was filtered through No. 42 Whatman filter paper. The extract was freeze-dried below -40°C, and the residues were redissolved in 10 ml of 80% aqueous methanol (HPLC grade) (J. T. Baker, USA), filtered through a 0.45 μm nylon membrane filter (TITAN, USA). The 20 μl filtrate was loaded on the HPLC system, a Shimadzu SPD-M10A HPLC system with a photodiode array detector (Japan) equipped with a Midas autoinjector. Separation was achieved on a 250 mm × 4.6 mm i.d., 5 μm, YMC-Pack ODS AM-303 (YMC) column. The absorbance of each sample solution was measured at 280 nm. The mobile phase was distilled water with 0.1% glacial acetic acid (solvent A) and acetonitrile with 0.1% glacial acetic acid (solvent B). The gradient was 0 min, 92% A; 0-2 min, 90% A; 2-27 min, 70% A; 27-50 min, 10% A; 50-51 min, 0%A; 51-60 min, 0% A; 60-63 min, 92% A. Run time was 60 min using a flow rate of 1 ml/min. The 30 standards and all solvents used (J. T. Baker) were of HPLC grade.

### Cell Culture

RAW 264.7 macrophage cells were obtained from the American Type Culture Collection (ATCC; USA) and maintained in Dulbecco’s Modified Eagle Medium (DMEM, Hyclone, USA) supplemented with 10% fetal bovine serum (FBS), penicillin (100 U/ml) and streptomycin (100 μg/ml) at 37°C and 5% CO_2_ condition.

### MTT Assay for Cell Viability

Cytotoxicity of CEFV on RAW 264.7 cells were determined by reduction of 3-(4, 5-dimethylthiazol-2-yl)-2,5-diphenyltetrazolium bromide (MTT) to formazan. Briefly, Raw264.7 cells were seeded in 96-well plates at a density of 1×10^4^ cells/well and were treated with various concentrations of CEFV (0-200 mg/ml). After 24 h of incubation, 100 ml of MTT reagent (5 mg/ml) was added to each well. After 4 h of incubation at 37°C, the medium was discarded and the formazan precipitates that formed in the live cells were dissolved in 100 ml of DMSO and the amount of formazan salt was determined by measuring the optic density (O.D) at 570 nm using an ELISA plate reader (Bio-Rad, USA). Cell viability was quantified as a percentage compared to the control.

### Assay for NO Production

The nitrite accumulated in culture medium was measured as an indicator of NO production based on the Griess reaction. Briefly, 100 ml of cell culture medium was mixed with 100 ml of Griess reagent [equal volumes of 1% (w/v) sulphanilamide in 5% (v/v) phosphoric acid and 0.1% (w/v) naphtylethylenediamine–HCl], incubated at room temperature for 10 min, and then the absorbance at 540 nm was measured in a microplate reader (Bio-Rad). NaNO_2_ was to as a standard.

### Assay for PGE_2_ Production

PGE_2_ level was quantified using enzyme immunoassay (EIA) kits according to the manufacturer’s instructions (Cayman Chemical, USA). Concentration ranges of 2.5 to 1 ng/well was used to construct the standard curve. PGE_2_ concentrations in the samples were calculated by using nonlinear regression of a four parameters logistic model.

### Total RNA Isolation and Reverse Transcription-polymerase chain reaction (RT-PCR)

RAW 264.7 cells were pretreated with various CEFV concentrations (0, 20, 40, 80 mg/ml) for 30 min and stimulated with LPS. After incubation for 12 h, total RNA was prepared using Trizol solution (Invitrogen, UK) according to the manufacturer's instructions. Reverse transcription and cDNA amplification were carried out with 1 mg of isolated total RNA using the RT-PCR kit (Clontech, USA). PCR analyses were performed on aliquots of the cDNA preparations to detect gene expressions of iNOS, COX-2 and b-actin, as internal standard. The primer sequences used for RT-PCR are as follows. iNOS forward 5’-CCT TGT TCA GCT ACG CCT TC-3’; reverse 5’-CTG AGG GCT CTG TTG AGG-3, COX-2 forward 5’- TGA AGC CCA CCC CAA ACA CAG T-3’; reverse 5’- TGA ACC CAG GTC CTC GCT TAT GAT-3’; β-actin forward 5’-TCA TGA AGT GTG ACG TTG ACA TCC GT-3’; reverse 5’-CCT AGA AGC ATT TGC GGT GCA CGA TG-3’. And quantitative real time PCR were performed using SYBR (Takara, Japan) with ABI PCR system (Applied Biosystems, USA). The Ct valued was calculated by subtracting the average Ct value of Actin from the Ct value of the target gene for each sample. The relative fold change of the experimental sample compared to the control was then calculated using the 2ΔΔCt method. The primer sequences used for qPCR are as follows. iNOS forward 5’-CCT CCT CCA CCC TAC CAA GT -3’; reverse 5’-CAC CCA AAG TGC TTC CAG TCA-3, COX-2 forward 5’-AAG ACT TGC CAG GCT GAA CT-3’; reverse 5’- CTT CTG CAG TCC AGG TTC AA-3’; β-actin forward 5’- GGC TGT ATT CCC CTC CAT CG-3’; reverse 5’- CCA GTT GGT AAC AAT GCC ATG T-3’.

### Western Blot Analysis

RAW 264.7 cells were pretreated with various CEFV concentrations (0, 20, 40, 80 mg/ml) for 30 min and stimulated with LPS (500 ng/ml) for 24 h. To detect protein phosphorylation levels, cells were pre-treated for 1 h with various concentrations of CEFV and stimulated with LPS (500 ng/ml) for 30 min. Cell pellets were lysed in 100 ml of cold RIPA buffer (Pierce, USA) containing protease and phosphatase inhibitor cocktail. Whole cell lysates which contained 20 mg of protein per lane, were separated on SDS-PAGE using a 10% resolving and 3%acrylamide stacking gel, and then transferred to nitrocellulose membrane (Millipore, USA) in a Western blot apparatus (Bio-Rad). The membranes were incubated overnight with blocking solution (5% skimmed milk in phosphate-buffered saline containing 0.05% Tween-20) at 4°C, and then with primary antibodies including anti-iNOS, anti-COX-2, anti-MAPKs for 16 h at 4°C. After washing twice with Twin 20/Tris-buffered saline (TTBS), membranes were immunoblotted with a 1:1000 dilution of horseradish peroxidase (HRP)-conjugated secondary anti-IgG antibody for 1 h at room temperature. Bound antibodies were detected using an enhanced chemiluminescence kit (Amersham Biosciences, UK). Equal loading was assessed using anti-GAPDH antibody to normalize the amount of total protein.

### Luciferase Activity Assay

RAW 264.7 cells grown in 24-well culture plates were co-transfected with 0.5 μg of NF-kB-dependent luciferase reporter plasmid pGL2×NF-κB (Kwon *et al*., 2010) and 50 ng of pRL-TK as the control *Renilla* luciferase vector (Promega, USA), using 1 ml Lipofectamine 2000 (Invitrogen). The cells were pretreated with various CEFV concentrations (0, 20, 40, 80 mg/ml) for 30 min and stimulated with LPS (500 ng/ml) for 24 h. Cells were harvested and treated with passive lysis buffer (Promega). Firefly and *Renilla* luciferase activities were measured using the Dual-Luciferase Reporter Assay System (Promega), according to the manufacturer’s instructions, and a GloMax™ 20/20 luminometer (Promega). Firefly luciferase activity of the reporter plasmid was normalized to *Renilla* luciferase activity. Independent triplicate experiments were performed.

### Statistical Analysis

Data were expressed as the mean ± SD values of three independent determinations. The statistical significance of all data was evaluated using the student’s *t*-test and one-way ANOVA followed by Duncan's multiple comparisons test. Differences of *p* < 0.05 were considered statistically significant.

## Results

### Effects of Fermented Extracts on Cytotoxicity and LPS-Induced NO Production

**Fig. 1:**
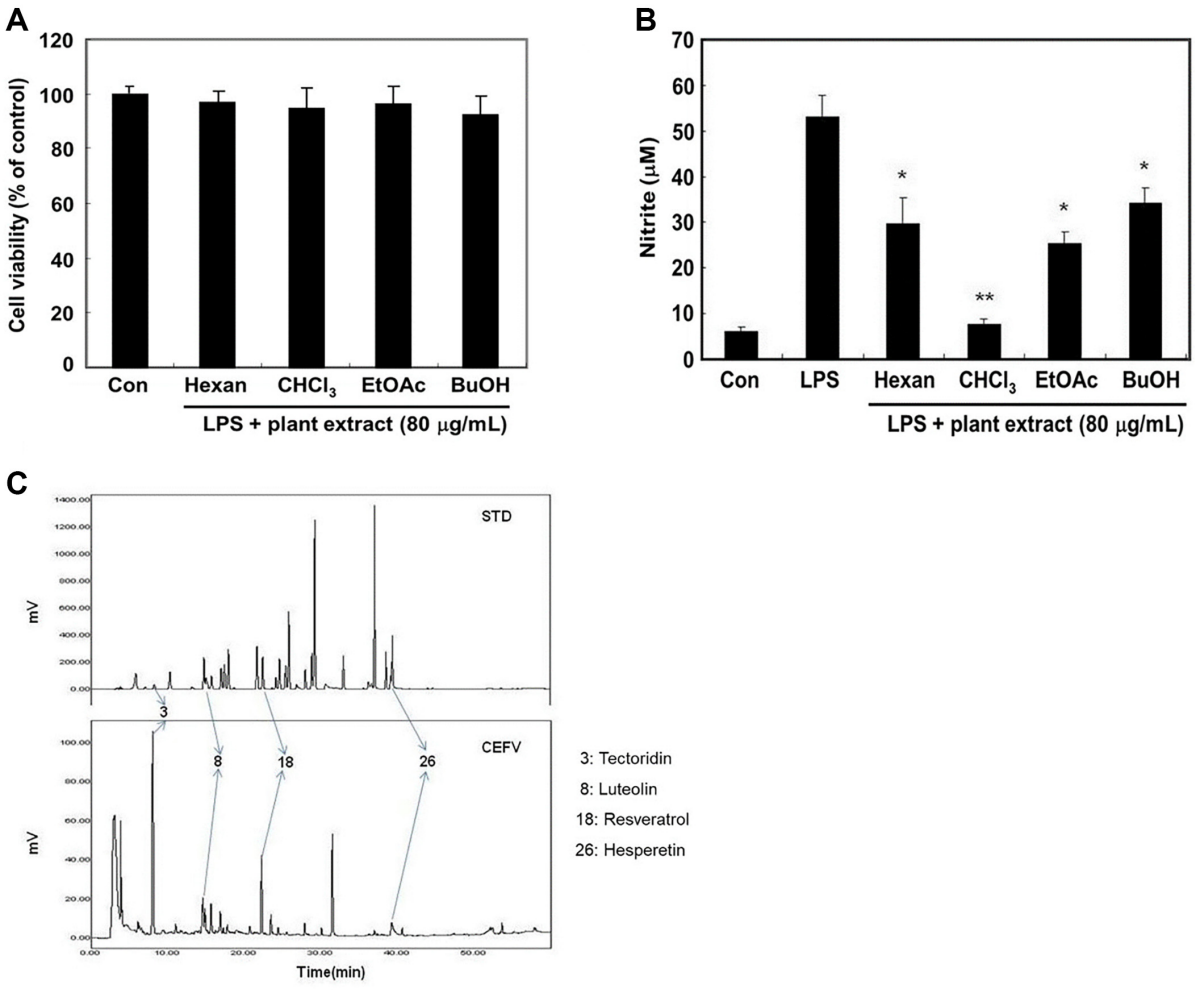
Effects of pre-treatment with four different fractions extracted from fermented *Viola mandshurica* and comparison of HPLC chromatograms of standard phenolic compound (STD) and CEFV. (**A**) Cell viability was measured using MTT assay 24 h after reagent treatment. (**B**) The production of NO was measured by using the method of Griess. (**C**) STD: 29 phenolic compound standards, 1. Gallic acid; 2. Pyrogallol; 3. Tectoridine; 4. Protocatechuic acid; 5. Gentisic acid; 6. Chlorogenic acid; 7. *p*-Hydroxybenzoic acid; 8. luteolin; 9. Caffeic acid; 10. Syringic acid; 11. Vanillin; 12. *p*-Coumaric acid; 13. Ferulic acid; 14. Veratric acid; 15. m-Coumaric acid; 16. Benzoic acid; 17. *O*-Coumaric acid; 18. Resveratrol; 19. *t*-Cinnamic acid; 20. β-Resorcylic acid; 21. Rutin; 22. Hesperidin; 23. MyricetinG; 24. Quercetin; 25. Naringenin; 26. Hesperetin; 27. Formononetin; 28. Biochanin A; 29. Naringin. Values are the mean ± SD of duplicated determinations from three separated experiments. *p < 0.05 and **p < 0.01 compared to LPS alone.

Fermented *V. mandshurica* extract was fractionated according to polarity (Hexan, CHCl_3_, EtOAc, or BuOH) and the cytotoxicity was evaluated in presence or absence of LPS (500 ng/ml) using MTT assay in RAW264.7 cells. All the fractions in presence of LPS did not affect the cell viability at 80 μg/ml (Fig. 1A). Next, to investigate the potential anti-inflammatory prosperities of four different fractions, we measured the production of nitrite, the stable product of NO. Raw264.7 cells were treated with 80 μg/ml concentration of four different fermented *V. mandshurica* fractions with LPS for 24 h. NO production was quantified utilizing the Griess reagent method. Among them, the chloroform extract (CHCl_3_) showed maximal inhibitory effect (85.8%) compared to LPS alone treatment on NO production (Fig. 1B). Therefore, we used the chloroform extract of *V. mandshurica* (CEFV) for further investigations.

### HPLC Profiles of CEFV

For the analysis of the main phenolic components in CEFV, twenty-nine standard phenolic compounds (STD) were employed to compare their retention times (RT) through HPLC analysis. Based on HPLC retention time, as shown in Fig. 1C, four peaks equivalent to STD were identified in CEFV and were confirmed to be tectoridin, luteolin, resveratrol and hesperetin.

### Effects of CEFV on NO Production, iNOS Protein and mRNA Expression in LPS-Induced RAW264.7 Cells

**Fig. 2:**
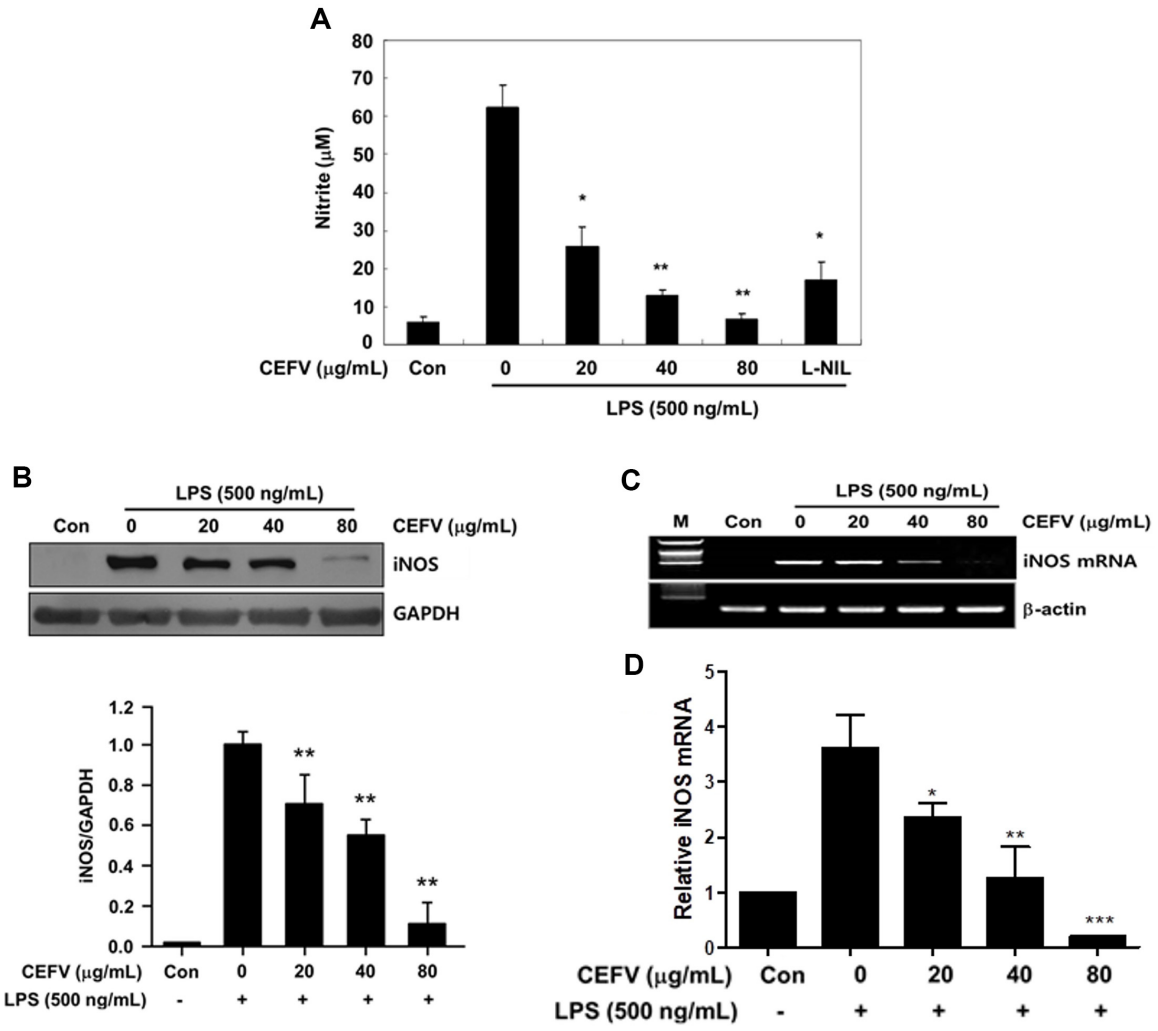
Effect of CEFV on LPS-induced nitric oxide (NO) production and inducible NO synthase (iNOS) expression in RAW264.7 cells. (**A**) Raw264.7 cells were pre-treated with indicated concentrations of CEFV for 30 min before being incubated with LPS (500 ng/ml) for 24 h. The culture supernatants were subsequently isolated and analyzed for nitrite levels. (**B**) The level of iNOS protein was detected 24 h after treatment of cells with LPS (500 ng/ml) with or without CEFV. Equal loading of proteins was verified by GAPDH. (**C**) Cells were pre-treated with indicated concentrations of CEFV for 30 min before being incubated with LPS (500 ng/ml) for 8h. iNOS specific sequences (499 bp) were detected by agarose gel electrophoresis. PCR of β-actin (285 bp) was performed in parallel to confirm equivalency of cDNA preparation. (**D**) Bar graph shows the qPCR results of iNOS. The values represent the mean ± SD for three independent experiments with triplicate measurements. *p < 0.05 and **p < 0.01 compared to LPS alone.

To explore the effect of CEFV on NO production, we pre-treated CEFV to sub-confluence cells for 30 min before being stimulated with LPS (500 ng/ml). After 24 h incubation, NO production in culture medium was quantified. The treatment of LPS alone induced approximately 11-fold greater NO production compared with LPS-untreated control, and this induction by LPS was significantly inhibited by CEFV treatment in a dose-dependent manner (Fig. 2A). Next, we treated iNOS inhibitor, which play an important role in NO production. CEFV at 40 μg/ml showed a stronger inhibitory effect than iNOS-specific inhibitor L-NIL (10 μM). In addition, the expression of the iNOS protein, responsible for catalyzing the production of NO from L-arginine, was scarcely detectable in unstimulated cells. However, its expression significantly increased in cells treated with LPS. This induction was significantly inhibited by CEFV in a dose-dependent manner (Fig. 2B). To assess the impact of CEFV on iNOS mRNA expression, cells were pre-treated with different concentrations of CEFV for 30 min before being stimulated with LPS for a duration of 12 h. Consistent with protein level, treatment with CEFV significantly inhibited LPS-stimulated iNOS mRNA expression in a dose-dependent fashion (Fig. 2C). Also, the qPCR results verified that the mRNA expression of iNOS was reduced (Fig. 2D). Thus, these results suggest that this mechanism contributes to the anti-inflammatory effects of CEFV, and reduction in nitrite production by CEFV treatment is mediated by transcriptional regulation of iNOS.

### Effects of CEFV on PGE_2_ Production and COX-2 Expression in LPS-Induced RAW264.7 Cells

**Fig. 3:**
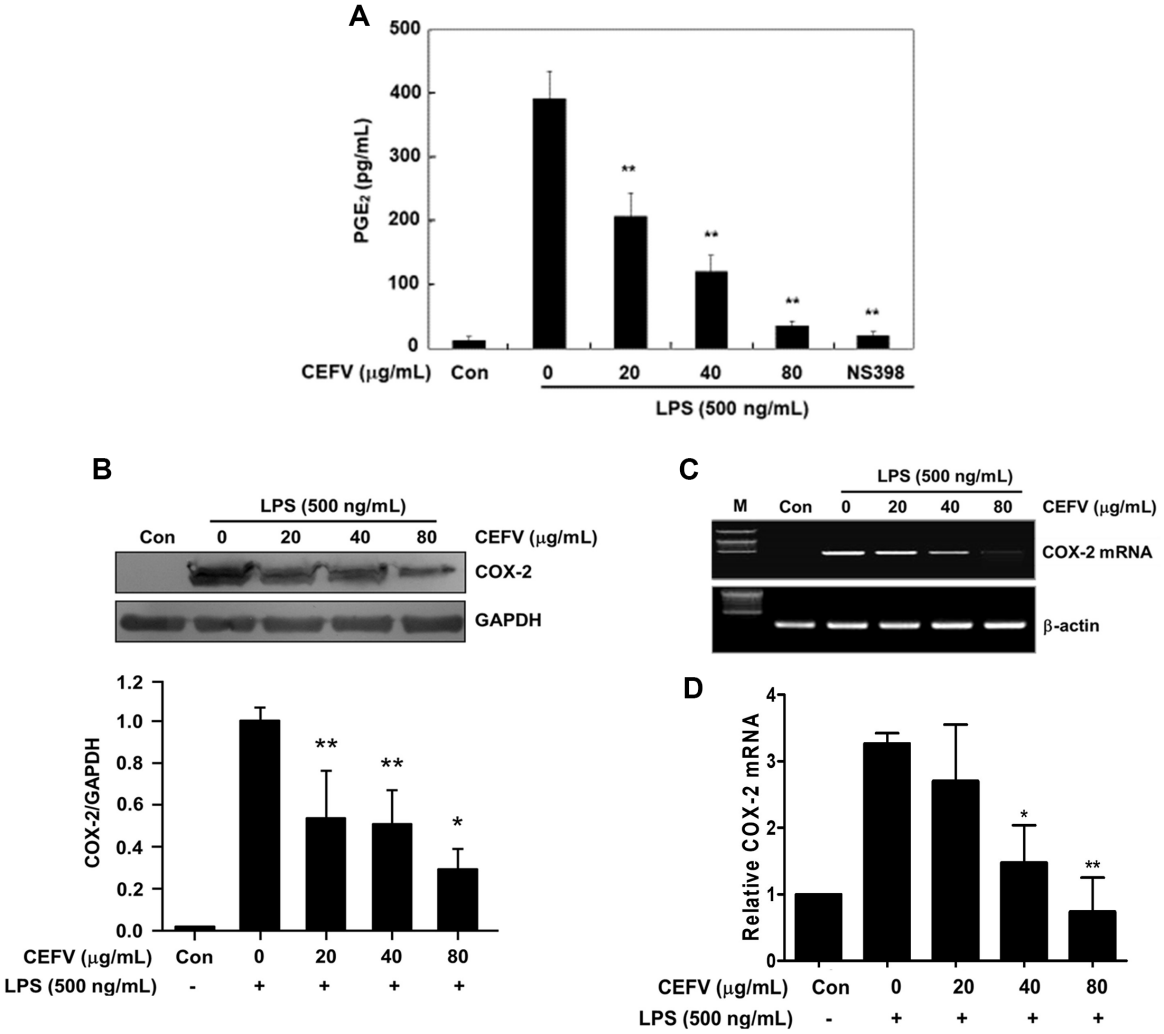
Effect of CEFV on LPS-induced prostaglandin E_2_ (PGE_2_) production and cyclooxygenase-2 (COX-2) expression in RAW264.7 cells. (**A**) Cells were pretreated with indicated concentrations of CEFV for 30 min before being incubated with LPS (500 ng/ml) for 24 h. PGE_2_ production was measured in cells pre-treated with indicated concentrations of CEFV for 30 min before being incubated with LPS (500 ng/ml) for 24 h. Control cells were incubated with vehicle alone. (**B**) The level of COX-2 protein was detected 24 h after treatment with LPS (500 ng/ml) with or without CEFV. Equal loading of proteins was verified by GAPDH. Bar graph shows densitometry analysis of COX-2/GAPDH ratio. (**C**) Cells were pre-treated with indicated concentrations of CEFV for 30 min before being incubated with LPS (500 ng/ml) for 8 h. COX-2-specific sequences (420 bp) were detected by agarose gel electrophoresis. PCR of β-actin (285 bp) was performed in parallel to confirm equivalency of cDNA preparation. (**D**) Bar graph shows qPCR results of COX-2. The values represent the mean ± SD for three independent experiments with triplicate measurements. *p < 0.05 and **p < 0.01 compared to LPS alone.

PGE_2_ is a crucial inflammatory mediator generated through the conversion of arachidonic acid by cyclooxygenase (COX) [19]. To confirm whether the CEFV could inhibit PGE_2_ production, we evaluated the inhibitory effects of CEFV on PGE_2_ production and COX-2 expression in LPS- induced RAW264.7 cells. Cells were pre-treated with CEFV for 30 min, and then activated with LPS (500 ng/ml) for 24 h. The quantification of PGE_2_ production was performed in the culture medium of RAW264.7 cells exposed to LPS, both in the presence and absence of CEFV. This analysis utilized an enzyme immunoassay (EIA) kit specifically designed for PGE_2_ measurement. As shown in Fig. 3A, the production of PGE_2_ was substantially elevated by LPS, but this increase was significantly suppressed in a dose-dependent fashion by CEFV treatment. We further assessed the effect of CEFV on COX-2 gene expression induced by LPS in RAW264.7 cells. As shown in Fig. 3B, 3C, and 3D the expressions of COX-2 protein and mRNA were effectively suppressed by CEFV treatment in a dose-dependent fashion. Collectively, these results suggest that CEFV has an effective inhibitory impact on the production of NO and PGE_2_. This inhibition is attributed to the downregulation of both mRNA and protein expression levels of iNOS and COX-2.

### Effects of CEFV on LPS-Induced MAPK Activation in RAW264.7 Cells

**Fig. 4:**
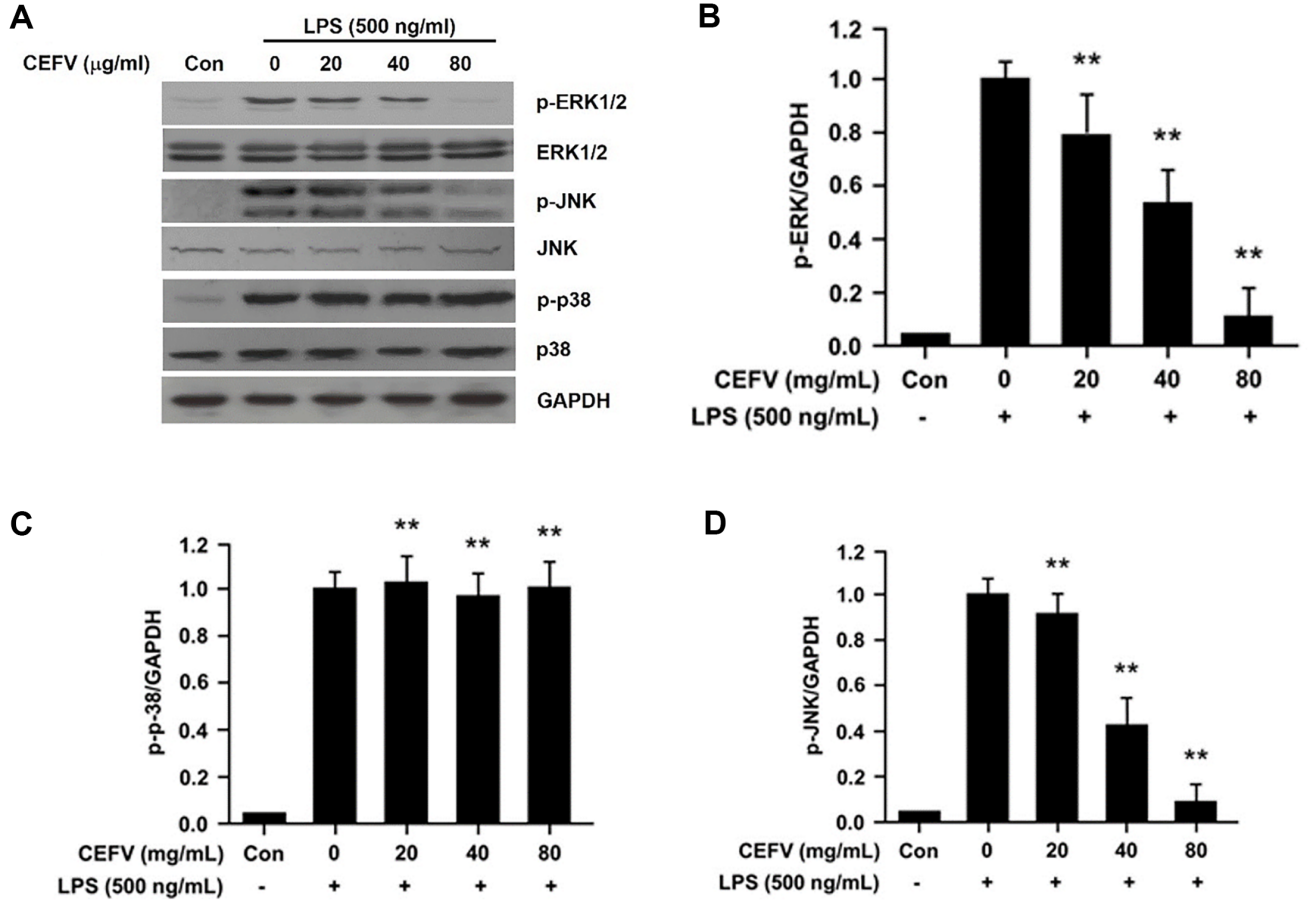
Effects of CEFV on LPS-induced MAPK signaling pathway in LPS-stimulated RAW264.7 cells. (**A**) Cells were pre-treated for 1 h with various concentrations of CEFV and stimulated with LPS (500 ng/ml) for 30 min and then analyzed by immunoblotting with antibodies against ERK, p38, JNK, p-ERK1/2, p-p38 and p-JNK. GAPDH was used as loading control of immunoblotting analysis. (**B-D**) Bar graphs show densitometry analysis of p-ERK1/2, p-p38 and p-JNK / GAPDH ratio. The values represent the mean ± SD for three independent experiments with triplicate measurements. **p < 0.01 compared to LPS alone.

Many studies have convincingly shown that LPS trigger MAPK activation, including ERK, JNK, and p38, resulting in the production of various inflammatory mediators, such as iNOS and COX-2 [4, 11, 20-22]. Thus, to explore the effects of CEFV on MAPK activation in LPS-induced RAW264.7 cells, cells were pre-treated with CEFV for 1 h, and then activated with LPS (500 ng/ml) for 30 min. MAPK activation by phosphorylation in LPS-induced RAW264.7 cells was investigated through Western blot analysis. As shown in Fig. 4, CEFV suppressed LPS-stimulated ERK and JNK activations in a dose-dependent way but did not affect p-38 activation. This result suggests that CEFV likely exerts its anti-inflammatory effects through repression of ERK and JNK signaling pathways in RAW264.7 cells.

### Effects of CEFV on LPS-Induced NF-κB Activation in RAW264.7 Cells

**Fig. 5:**
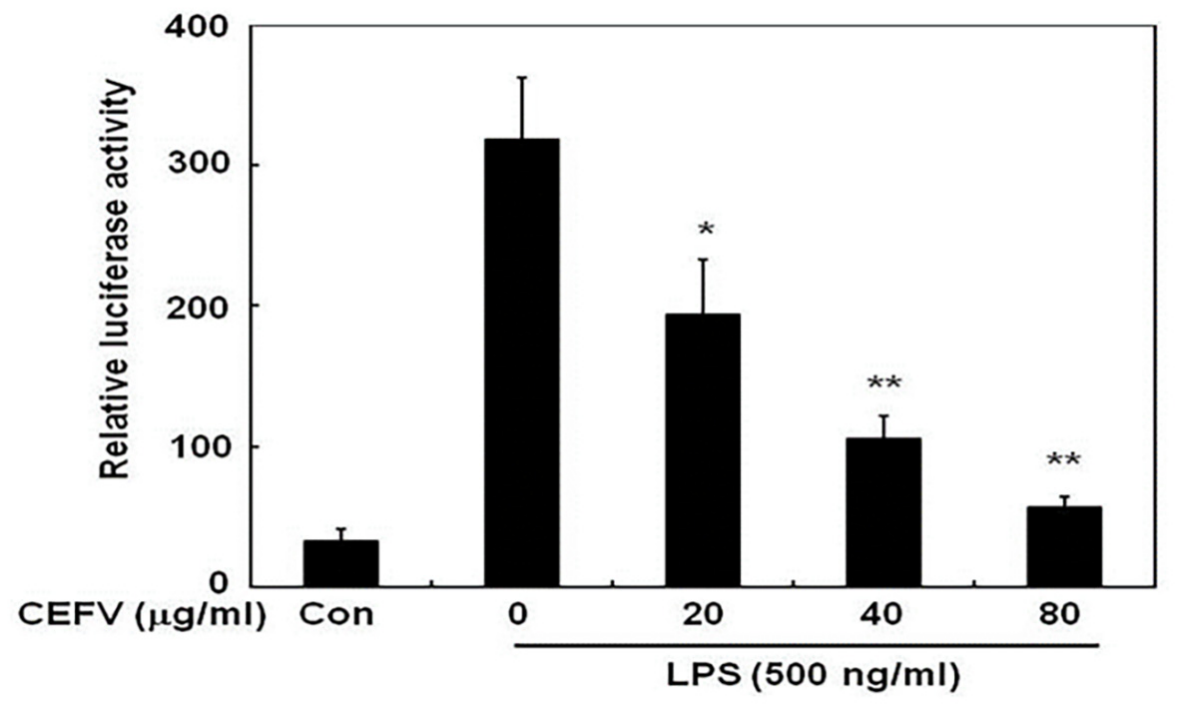
Effects of CEFV on NF-kB activation in LPS-stimulated RAW264.7 cells. (**A**) Luciferase assay. The NF-kBdependent reporter plasmid pGL2-3 x NF-kB was co-transfected into RAW264.7 cells with pRL-TK as the internal control. The transfected cells were pre-treated with indicated concentrations of CEFV for 30 min and then stimulated with LPS (500 ng/ml) for 24 h. All firefly activity was normalized to the *Renilla* luciferase activity derived from pRL-TK. The values represent the mean ± SD for three independent experiments with triplicate measurements. *p < 0.05 compared to LPS alone. *p < 0.05 and **p < 0.01 compared to LPS alone.

LPS-induced NF-κB activation plays a significant role in modulating the expression of inducible iNOS and COX-2 genes during inflammation [4, 11, 20-22]. To investigate the impact of CEFV on NF-κB activation by LPS stimulation in RAW264.7 cells, the pGL2-3 × NF-κB-luciferase plasmid [23], which harbors three consecutive repeats of the NF-κB binding motif along with the luciferase gene, was used for the NF-κB transactivation assay. As shown in Fig. 5, a remarkable elevation in luciferase activity was found after LPS stimulation, suggesting that NF-κB activation was induced by LPS. This NF-κB activation by induced LPS was inhibited in a concentration-dependent fashion through pre-treatment with CEFV (20, 40, 80 μg/ml) for 30 min. This result indicates that CEFV inhibited NF-κB activation induced by LPS in RAW264.7 cells.[Fig F6]

## Discussion

In this study, we have illustrated that chloroform extract from fermented *Viola mandshurica* (CEFV) exhibits anti-inflammatory activity in LPS-stimulated RAW264.7 macrophage cells. Moreover, we have also elucidated the molecular mechanism underlying the anti-inflammatory activity of CEFV. It is well known that LPS induces transcription and translation of iNOS and COX-2, which are responsible for the production of pro-inflammatory mediators NO and PGE_2_, respectively, in RAW264.7 cells [4, 11, 20-22]. Both iNOS and COX-2 are essential components of the inflammatory response and their products, NO and PGE_2_, respectively, contribute to the various physiological changes associated with inflammation. Therefore, their regulation and modulation are important targets for therapeutic interventions aimed at controlling inflammation in various diseases and conditions. Furthermore, we found that CEFV inhibited NO and PGE_2_ in dose-dependent patterns in LPS-stimulated RAW264.7 cells without notable cytotoxicity, as shown in MTT assay. Furthermore, CEFV exhibited a dose-dependent suppression of iNOS mRNA and protein expression levels. Similar to NO and iNOS, CEFV also decreased LPS-induced PGE_2_ production as well as COX-2 expression in mRNA and protein levels in RAW264.7 cells. These findings suggest that CEFV represses LPS-induced productions of NO and PGE_2_ by downregulating iNOS and COX-2 expressions, respectively.

In recent years, it has become clear that the expression of inducible iNOS and COX-2 genes are upregulated by activation of transcription factor NF-κB and MAPK signaling pathways in RAW264.7 cells stimulated by [4, 11, 20-22]. Accumulating evidence suggests that blocking activation of NF-κB and MAPK pathways by numerous plant extracts can remarkably suppress the production of NO and PGE_2_, along with iNOS and COX-2 expression in RAW264.7 cells stimulated by LPS [4, 11, 20-22]. In this study, we demonstrated that CEFV effectively inhibits LPS-induced NF-κB transcription in a dose-dependent manner. Collectively, our results suggest that the suppression of iNOS and COX-2 expression at both mRNA and protein levels by CEFV is likely attributed to the inhibition of NF-κB.

Numerous prior studies have demonstrated that LPS induces the phosphorylation-dependent activation of MAPK, including p38, ERK, and JNK, in RAW264.7 cells. These phosphorylation events are essential for the NF-κB activation in response to LPS [4, 11, 20-22]. In this study, we observed that CEFV dose-dependently inhibited the activation of ERK and JNK, while showing no significant impact on p38 activation. This suppression was achieved by attenuating LPS-induced phosphorylation in RAW264.7 cells. These findings are consistent with previous studies [22] demonstrating that various compounds or extracts exhibit anti-inflammatory effects by blocking NF-κB, ERK, and JNK signaling pathways in LPS-induced RAW264.7 cells. Taken together, our results suggest that CEFV hinders the production of key pro-inflammatory molecules, namely NO and PGE_2_, through the downregulation of iNOS and COX-2 expression. This effect is achieved by suppressing NF-κB, ERK, and JNK signaling pathways in LPS-induced RAW264.7 cells.

HPLC analysis revealed that the major constituents in CEFV are tectoridin, luteolin, resveratrol and hesperetin. Among them, anti-inflammatory actions and mechanisms of luteolin [24, 25], resveratrol [26, 27] and hesperetin [28, 29] have been extensively studied and their contents are well reviewed. In addition, tectoridin has been recently reported to alleviate LPS-induced inflammation in RAW264.7 cells [30]. Considering these findings, it is presumed that the four main components in CEFV primarily contribute to the anti-inflammatory activity in LPS-induced RAW264.7 cells. On the other hand, because it is known that contents of natural fermentation products can vary considerably depending on the conditions applied to the fermentation process, such as the fermentation period, fermentation temperature and the fermentation method [9-11], further studies are needed to determine the most suitable fermentation conditions to produce bioactive components with better anti-inflammatory effects from CEFV.

In conclusion, this study demonstrated that CEFV possesses anti-inflammatory effects by downregulating LPS-induced iNOS and COX-2 expressions, leading to decreased production of pro-inflammatory mediators NO and PGE_2_, respectively, via ERK-, JNK- and NF-kB-dependent pathways in RAW264.7 cells, and that CEFV can be used as a protective agent against inflammation. Further study will be needed to investigate which bioactive components in CEFV exhibit anti-inflammatory effects. In addition, an in vivo study of CEFV using animal models of inflammation or clinical trials will help in the development of new natural herb-derived medicinal drugs for the treatment of anti-inflammatory diseases.

## Figures and Tables

**Fig. 6 F6:**
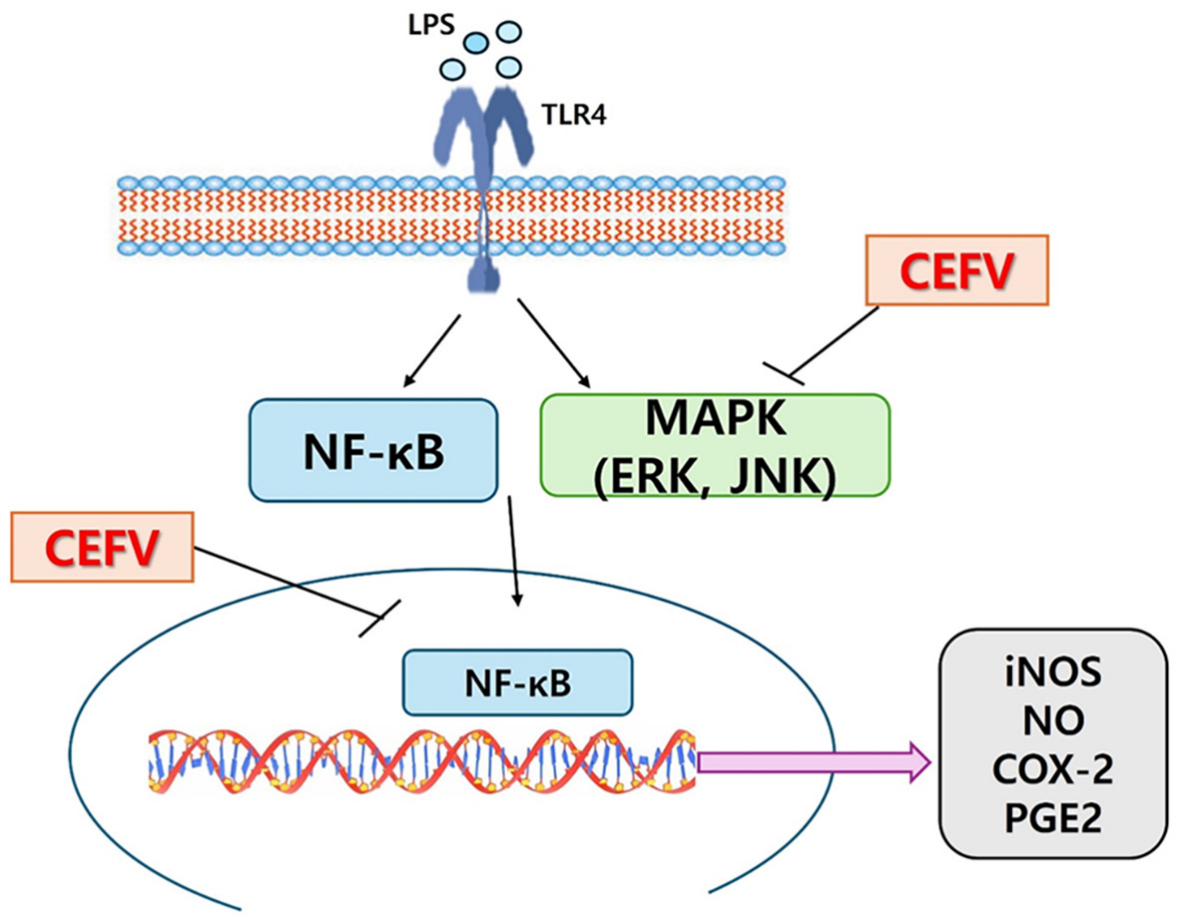
Chloroform extract from fermented *Viola mandshurica* (CEFV) regulates LPS-induced inflammation response in RAW 264.7 cells.
